# Enrichments of Cadmium and Arsenic and Their Effects on the Karst Forest Area

**DOI:** 10.3390/ijerph16234665

**Published:** 2019-11-22

**Authors:** Jinfeng Wang, Xiaoyong Bai, Fang Liu, Jian Zhang, Fei Chen, Qian Lu

**Affiliations:** 1 College of Resource and Environmental Engineering, Guizhou University, Guiyang 550025, China; jfwanggz@126.com (J.W.); jzhanggzdxhjkx@163.com (J.Z.); chenfeicn@126.com (F.C.); lq985479226@163.com (Q.L.); 2 State Key Laboratory of Environmental Geochemistry, Institute of Geochemistry, Chinese Academy of Sciences, Guiyang 550081, China; Baixiaoyong@126.com; 3 School of Tourism, Historical Culture, Liupanshui Normal University, Liupanshui 553004, China; 4 Puding Karst Ecosystem Observation and Research Station, Chinese Academy of Sciences, Puding 562100, China

**Keywords:** cadmium, arsenic, physicochemical properties of soil, correlation analysis, karst forest area

## Abstract

An understanding of the enrichment mechanisms of cadmium (Cd) and arsenic (As) in the process of rock weathering and soil formation is essential to develop agriculture according to local conditions. However, the enrichments of soil Cd and As under natural background conditions in karst areas are still uncertain. The enrichment factor, geo-accumulation index, redundancy analysis, and other methods were used to analyze the enrichment degree and the influencing factors of Cd and As on 5 rock–soil profiles and 15 topsoil samples, which were collected from a karst forest area in Libo County, Guizhou Province. The results showed that the enrichment process was divided into three stages. In the first stage, Cd and As were enriched in carbonate rocks, and their mean concentrations were 1.65 and 3.9 times those of the corresponding abundance of the crust. In the second stage, the enrichment of the parent rock into the soil, the enrichment factors of Cd and As in the parent material horizon relative to the bedrock horizon were 9.2 and 2.82, respectively. The third stage refers to the enrichments of Cd and As in the topsoil, where Cd enrichment was more obvious than that of As. Soil organic matter (SOM) and phosphorus (P) are important factors that influenced the enrichments of Cd and As in the topsoil. The functional groups of SOM were complexed with Cd and As; P easily formed precipitates with Cd, and the tree litter was fed back to the topsoil, which may be the reason for the surface enrichment of Cd and As. This study will help the scientific community understand the enrichment mechanisms of soil Cd and As in karst areas.

## 1. Introduction

Cadmium (Cd) frequently co-exists with arsenic (As) in soils [1]. The enrichments of Cd and As have always been an important scientific issue for environmental ecosystems. The karst area in southwest China is considered a geochemically anomalous area of Cd and As, with Guizhou province in the center. The mean Cd and As concentrations in the soil for this region were 0.37 mg/kg [2] and 19.82 mg/kg [3], respectively. These values were higher than the mean concentrations of Cd (0.27 mg/kg) and As (11.2 mg/kg) in China and considerably higher than the worldwide mean concentrations of 0.36 mg/kg and 6.0 mg/kg, respectively [4,5]. Cadimum and As are non-essential for the human body and are extremely dispersed potentially toxic elements (PTEs) in soils [6,7,8]. High concentrations of Cd and As in soils can seriously affect the growth and development of plants. Cadimum and As can also enter the human body through the food chain, which may affect human health [9,10,11,12,13]. The enrichment of Cd and As in soils, and their resulting ecological risks and potential threats to human health, have attracted widespread attention from local and foreign scholars.

Several studies have suggested that the enrichment of Cd and As in karst areas is due to the high geochemical background values of Cd and As in the soil [14,15]. A series of studies proposed that the enrichment of Cd and As is closely related to carbonate rocks [16,17], which have high contents of Cd and As. Other scholars have argued that a double enrichment mechanism is the source of Cd and As in karst areas. A notable change of rock/soil volume during carbonate rock weathering can easily lead to the relative enrichment of PTEs, such as Cd and As, from rock to soil [18]. In the process of rock weathering, the soil inherits the high background geochemical characteristics of the parent rock, which may form the high background values of Cd and As concentrations in the soil.

Some progress has been made in understanding the sources and enrichment characteristics of Cd and As in the soil. However, the specific enrichment process and formation mechanism of PTEs in karst remain unclear. Second, the many previous studies have only focused on topsoil; thus, research on PTE enrichment of the entire rock–soil profile is lacking. Third, the majority of studies focused on agricultural areas cultivated by humans, polluted industrial areas, and mining areas, and less on forest areas where human activities have had a weak impact. Based on these issues, this study takes a karst forest in Libo County, Guizhou Province, as its study area to explore the enrichment process of Cd and As in the soil throughout the rock–soil profile and its influencing factors on the karst forest ecosystem. Our findings provide a scientific basis for the spatial distribution and enrichment of Cd and As in the process of rock weathering and soil formation in karst areas.

## 2. Materials and Methods

### 2.1. Study Area and Samples Collection

**Figure 1:**
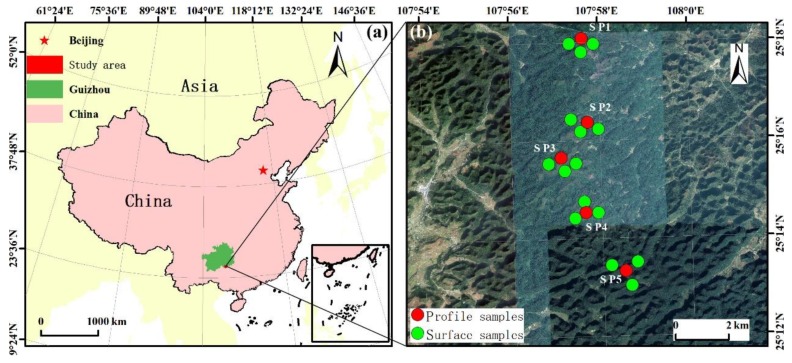
Geographical location and sampling point distribution in the study area.

The study area is located near the Maolan National Natural Reserve in Libo County, southeastern Guizhou, with a geographical location of 107°54′–108°02′ E, 25°12′–25°18′ N. This area is predominated by Carboniferous and Permian carbonate rocks. This forest area is also affected by anthropogenic activities to a lesser degree. The soil was mainly formed by weathering through limestone, dolomite, and argillaceous carbonate rocks. The study area is located in a mid-subtropical monsoon climate zone, with an average annual temperature of 15.3 °C and an annual precipitation of 1752.5 mm, mainly from April to October. The natural vegetation of the area is evergreen broad-leaved forest. In October 2018, we conducted field sampling and chose the representative sections in the karst mountainous areas, as well as in secondary forests, to excavate the soil profile. According to the principle of soil layering, the soil profile can be divided into the humus layer (Horizon A), the leached layer (Horizon B), the parent material layer (Horizon C), and the bedrock layer (Horizon D). A total of five soil profiles were collected, and three surface soil samples close to each profile were randomly taken at 0–15 cm (Figure 1). Surface soil samples were taken by the plum-shaped point method. Concretely, approximately 1.0 kg of topsoil samples were collected at 5 points, including the 4 vertices and the center of the (10 m × 10 m) square. After mixing, the samples were divided by quarters, and, finally, ~1.0 kg of soil samples were retained as the point. A total of 15 mixed surface soil samples were collected.

### 2.2. Determination of the Soil and Bedrock Samples

All soil samples were naturally air-dried for one month under room temperature conditions in the laboratory to remove grit and animal and plant residues. The samples were then ground and passed through a nylon sieve with a pore size of 0.25 mm. The bedrock was broken and treated in the same manner. Soil pH was determined using an acidity meter (HI98160, HANNA, USA) (water to soil ratio of 2.5:1), and soil organic matter (SOM) was determined by the potassium dichromate volumetric method [19]. Humic acid (HA) was determined by the NaOH and Na_4_P_2_O_7_ solution extraction and K_2_CrO_4_ solution oxidation methods [20]. Total phosphorus (P) was determined by the sulfuric acid–perchloric acid digestion–molybdenum blue colorimetric methods [21]. Soil total nitrogen (N) was determined by the Kjeldahl determination method [22]. Soil particle size distribution was analyzed using an international soil quality classification standard [23]. Thereafter, the 2 mm air-dried soil samples were divided into three types—clay, silt, and sand—using the hydrometer method.

The soil Cd was analyzed using inductively coupled plasma mass spectrometry (NexION300X, PerkinElmer, Waltham, MA, USA), and As was measured using an atomic fluorescence spectrophotometer (AFS-920, Beijing Titan Instruments Co. Ltd., China), with detection limits of 0.01 and 0.05 mg/kg for Cd and As, respectively. Approximately 100 mg of the soil samples were digested in a high-pressure Teflon vessel using a HF + HNO_3_ (ultra-pure, 0.5 mL +5 mL) mixture for 48 h (150 °C). Exactly 2 mL of the digests were diluted to 50 mL in deuterium depleted water for testing. The quality controls consisted of method blanks, certified reference materials (CRMs), and blind duplicates. The CRMs were in agreement with recommended values (with the recovery rate ranging from 92% to 110%), and the analytical precision was generally better than 5% for all elements.

### 2.3. Geo-Accumulation Index

In 1986, the German scientist Müller introduced the geo-accumulation index (Igeo) [24]. This method considers human factors, environmental geochemical background values, and the influence of natural diagenesis background values, which can be used to reflect the degree of PTE enrichment in soils or other materials. The calculation formula was presented as follows:

$$I_{geo}=\log_{2}\left(\frac{C_{i}}{kB_{i}}\right)$$

where *C_i_* is the measured concentrations of Cd or As, and *B_i_* is the geochemical background values of Cd and As. *k* is a constant, which considers that diagenesis may initiate a change in the background values (1.5 was frequently used). The background values of Cd and As in the surface soil of Guizhou were 0.31 and 14.89 mg/kg, respectively, and these background values are representative for Guizhou province [25]. Table 1 presents the enrichment degree corresponding to the Igeo.

### 2.4. Statistical Analysis

To evaluate the effect of the physicochemical properties of soil on the concentrations of Cd and As, we used the R3.5.3 software (MathSoft Company, Cambridge, Massachusetts, USA) to analyze the correlation among Cd, As, and the influencing factors. Pearson’s correlation is a statistical method for studying the correlation between random variables to reflect the closeness of the relationship between variables. The Kolmogorov–Smirnova and Shapiro–Wilk methods were used to test the normality of Cd and As contents in surface soil based on the IBM SPSS 20 software (IBM Corporation, Armonk, NY, USA). To further explore the relationship between the PTEs and the physicochemical properties of soil within a spatial distribution, a redundant analysis was performed using the Canoco 4.5 software (Microcomputer Power, Ithaca, NY, USA). In addition, to construct a stepwise regression model, we performed a stepwise regression analysis using IBM SPSS 20 software.

## 3. Results and Discussion

### 3.1. Distribution Characteristics of Cd and As in Rock–Soil Profiles

**Figure 2:**
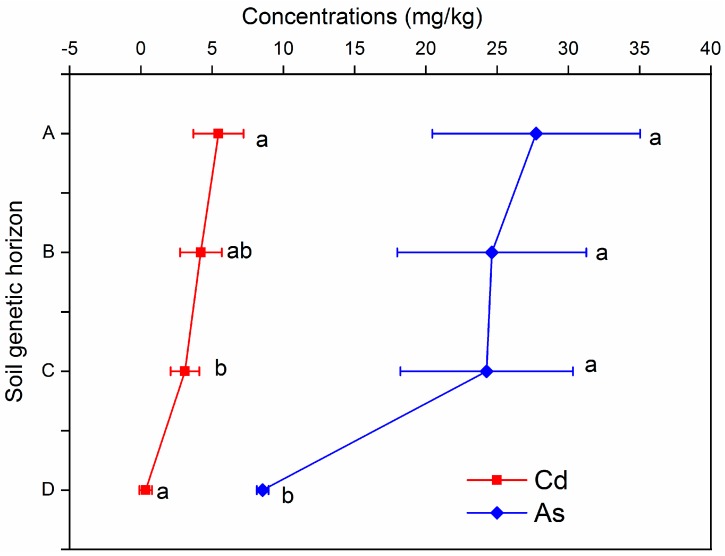
The distribution characteristics of Cd and As content across soil layers. The number of statistical samples per layer is 5. The bars are the mean standard deviation.

The Cd and As contents of the different layers of soil and bedrock in the rock–soil profiles of the study area were 5.44 and 27.74 mg/kg (horizon A) > 4.21, 24.62 mg/kg (horizon B) > 3.09, 24.26 mg/kg (horizon C) > 0.33, and 8.54 mg/kg (horizon D) (Figure 2).

In general, the Cd and As concentrations were maximal and minimal in Horizons A (surface soil) and D (lower bedrock), respectively. The mean concentrations of As and Cd in the limestone were 3.9 and 1.65 times the abundance of crust at 2.2 and 0.2 mg/kg, respectively [26]. This finding indicates that the geological background of the limestone in the study area has a high geochemical background of Cd and As. A previous study showed that the Cd concentration of braided carbonate rocks of the Middle Jurassic in Switzerland points to extreme enrichment, as the highest concentration of Cd was 4.9 mg/kg [27]. The Cd concentration of the Permian limestone in central Japan was relatively high, ranging from 0.17 to 2.41 mg/kg, with an average concentration of 0.5 mg/kg [28]. The results obtained in this study are similar to those of previous studies.

The Cd and As contents of the soil samples from layers A, B, and C far exceed the soil limit values (0.20 and 15 mg/kg) of soil environmental quality standards (GB15618-1995) in China. Furthermore, these values are higher than the background values for Cd and As in Guizhou soil [25], thereby indicating that the soil in the study area has high Cd and As geochemical backgrounds. The study showed that the Cd concentrations of soils in cultivated land (1.33 mg/kg) and forest land (1.57 mg/kg) in the karst distribution area of Luodian County, southern Guizhou, were significantly greater than those of non-karst rock areas (0.27 mg/kg) and woodland soil (0.22 mg/kg). The high concentration of Cd in the soil was clearly controlled by the distribution of carbonate rocks [29]. In a small area of southwest Guizhou, the concentration of As in agricultural land in the karst area (47.9 mg/kg) was also significantly greater than those in the sub-karst area (36.8 mg/kg) and in non-karst area (12.7 mg/kg) [30]. In addition, the concentrations of As in the overlying soil in Guizhou and carbonate rock were high, whereas the concentration of As in clastic rocks was low. Furthermore, the background value of carbonate rocks for As was high, and As easily produced relative enrichment in the process of rock weathering and soil formation. These are the main reasons for high As content in carbonate rock distribution areas [3,31]. Cadmium and As in soil developed by carbonate parent materials showed significant enrichment characteristics in southern Guangxi [32]. In summary, the Cd and As contents of the carbonated weathered soil profiles show a high background distribution, where the Cd and As enrichments of the topsoil are notable in forest areas weakly affected by human activities.

### 3.2. Enrichments of Cd and As Across Soil Layers

As mentioned, Cd and As exhibit relative enrichment characteristics in carbonate rocks relative to the crust, which provides a high background value for weathered soil.

**Figure 3:**
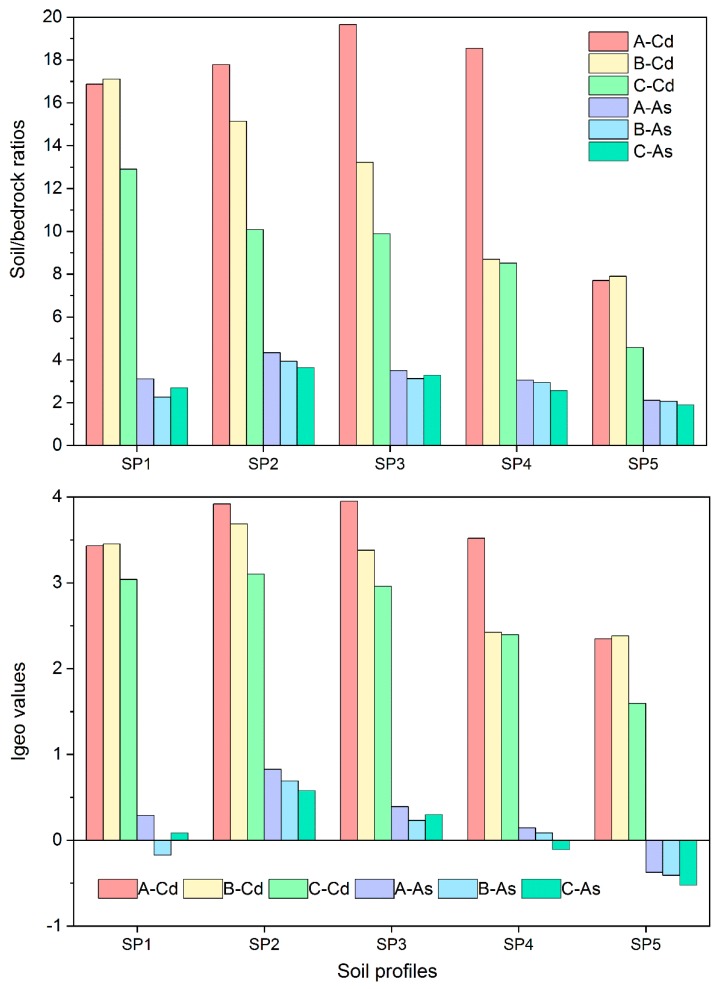
Geo-accumulation indexes of Cd and As content across soil layers and the bedrock ratios in five soil profiles.

The enrichment factor of Cd and As contents in the A, B, and C layers of the five soil profiles with respect to bedrock horizon (D) was calculated to explore the relative enrichment degrees of Cd and As during soil formation through the weathering of carbonate rocks (Figure 3). Figure 3 shows that the enrichment factor of Cd in Horizon C relative to Horizon D was between 4.58 and 12.9 with a mean value of 9.2, whereas that of As was between 1.9 and 3.64 with a mean value of 2.82. This finding indicates that Cd and As were relatively enriched and showed “second-stage enrichment” when Horizon C was formed by the weathering of carbonate rocks.

Cadmium was more enriched than As. Compared with Horizon C, Horizon B had an enriched Cd. In profiles one and five, the Cd concentration in Horizon B was slightly greater than that in Horizon A. On the other hand profiles two, three, and four showed that Horizon A was significantly greater than horizon B.

In soil profiles one and three, the concentration of As in Horizon B was slightly lower than that in Horizon C, whereas the opposite was true for profiles two, four, and five—that is, Horizon B was higher than Horizon C. The enrichment degree of As in Horizon A was higher than that in Horizons B and C. In summary, Cd and As in surface soil were relatively enriched with respect to their parent material layer, and the average relative enrichment factors were 1.78 and 1.14, respectively. Results indicate that Cd and As were further enriched during the development of humus (Horizon A) from the parent material layer (Horizon C).

The results of the Igeo method show that the soil in each layer of the five profiles has greater than moderate enrichment (Figure 3), with moderate to heavy enrichment accounting for 33.3% and heavy enrichment accounting for 60%. The ratio of As in the soil for each layer with non-enrichment to moderate enrichment was 66.7%, and the remainder pertained to non-enrichment. In summary, humus (Horizon A) is more concentrated than the parent material layer (Horizon C), and Cd and As are concentrated in the surface soil, which indicates “third-stage enrichment”.

### 3.3. Effects of the Physicochemical Properties of Topsoil on Cd and As Contents

**Figure 4:**
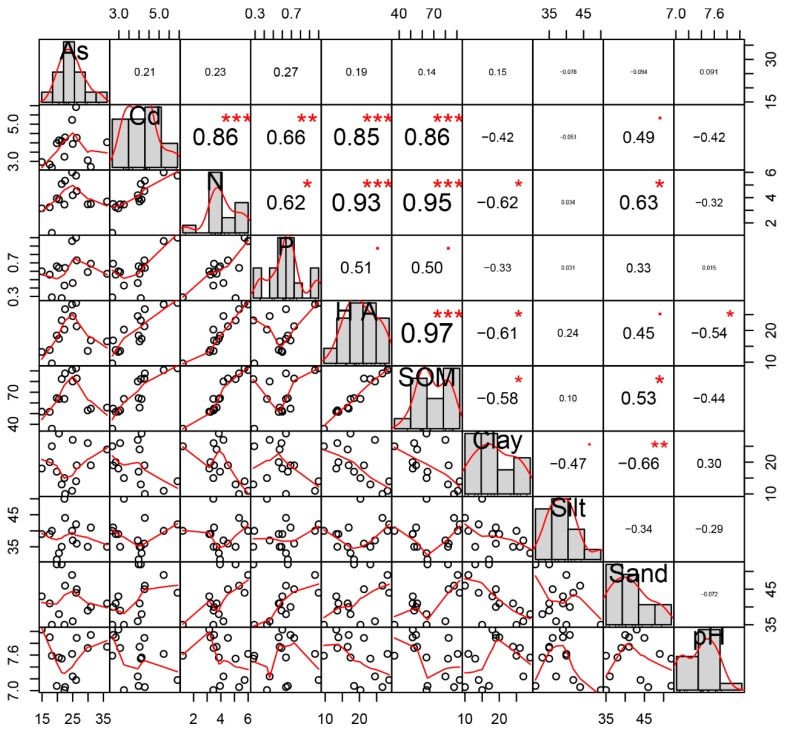
Correlation analysis of soil Cd, As, and soil physicochemical parameters (* manifest *p* < 0.05, ** manifest *p* < 0.01, *** manifest *p* <0.001).

**Figure 5:**
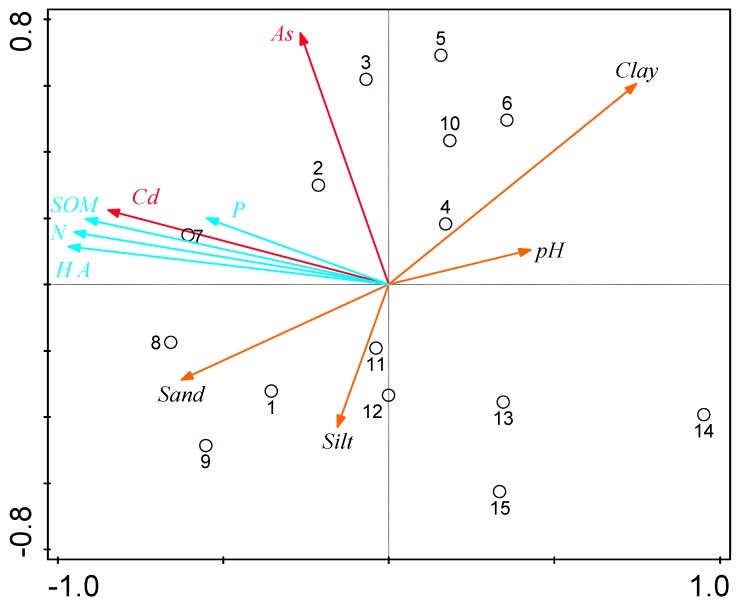
Bioplot map of hybrid redundancy analysis (RDA) for potentially toxic elements (PTEs) and environmental factors.

Cadmium and As show notable enrichment in Horizon A. Several studies have shown that PTEs exhibit surface aggregation in the soil profile [33,34]. Similarly, PTE enrichment and pollution mainly affect the topsoil. Therefore, the effects of the physicochemical properties of the surface on soil Cd and As enrichment were analyzed and are provided in Table 2, as well as Figure 4 and Figure 5.

The total concentrations of Cd and As in the surface soil ranged from 2.67 to 5.92 mg/kg (n = 15) and from 14.90 to 36.20 mg/kg, respectively. The contents of both Cd and As obeyed normal distribution, which is based on the Kolmogorov–Smirnova and Shapiro–Wilk of methods. The average concentration for Cd was 3.83 ± 0.93 mg/kg, while the mean value for As was 23.8 ± 5.6 mg/kg. The average concentrations of Cd and As in the topsoil were 12.4 and 1.6 times the background values of soil Cd and As in Guizhou, respectively. The average pH value was 7.54 ± 0.35, which is slightly alkaline. The SOM concentration reached an average value of 67.35 ± 17.39 g/kg. The mean values for total P and N concentrations were 0.61 ± 0.20 and 4.02 ± 1.20 mg/kg, respectively. Total HA concentration ranged from 9.61 to 28.65 mg/kg. The soil particle size distribution showed that the surface soil was composed of clay loam.

Figure 4 depicts the correlation analysis among the eight indicators of the physicochemical properties of the topsoil and concentrations of Cd and As. The figure shows that Cd in the topsoil was significantly positively correlated with N, P, HA, and SOM, with correlation coefficients of 0.86, 0.66, 0.85, and 0.86, respectively. Total Cd was positively correlated with sand, with a correlation coefficient of 0.49. The mineral composition of sand was the residual particles of limestone weathering. In addition, the mineral composition of the grains was mainly calcite with a chemical composition of mainly CaCO_3_. Cd could easily replace Ca in the form of an isomorphism and enter the crystal lattice of calcite minerals, which would be beneficial for the accumulation of Cd.

Second, the topsoil was alkaline; thus, the migration was weak, and Cd easily accumulated in the topsoil. However, the total Cd in the topsoil was negatively correlated with pH and the proportion of clay in the soil particulate distribution; the correlation coefficients were −0.42 and −0.38. It can be seen that the soil alkalinity and soil aggregate particulate distribution were not unique factors affecting PTE concentrations. At the same time, no significant correlation was observed between the total As and soil physicochemical parameters, thereby indicating that Cd was more likely to accumulate in the topsoil compared with As.

The results of redundancy analysis (RDA) showed a notable separation among the 15 sample points on the surface (Figure 5), which were distributed in the four quadrants and indicated that, although the soil type remained the same, the topsoil exhibited a certain degree of spatial heterogeneity.

Figure 5 shows that the concentrations of Cd and As in the topsoil were in the same quadrants as N, P, HA, and SOM. The direction of the arrow remains the same, and the angle between each factor is acute, which shows that surface soil concentrations of Cd and As were mainly influenced by N, P, HA, and SOM. In addition, the angle between the sand was acute, which indicates that it was also affected by the composition of sand in the soil. PTEs are present in the soil particles. Their amount in the soil has a considerable influence on the distribution and migration of heavy metals, which indicates that the concentration of heavy metals in soil was closely related to soil texture. In summary, the enrichment of Cd in surface soil was mainly influenced by SOM, N, HA, and P. As is mainly affected by SOM and P.

### 3.4. Regression Analysis

Studies have shown that correlation may not necessarily indicate a significant causal association. The stepwise regression method gradually selects variables according to their contribution values and obtains the important influencing variables by eliminating factors with non-significant contribution values. The regression equations for Cd and As constructed by the stepwise regression model were derived as follows:

Y_1_ = 6.298 + 1.679X_2_ + 0.032X_4_ − 0.037X_6_ − 0.566X (F = 14.78, *p* < 0.01; R = 0.925; Ry_1_ = 0.617, Ry_2_ = 0.761, Ry_3_ = −0.424, Ry_4_ = −0.41; t_1_ = 2.477, t_2_ = 3.711, t_3_ = 1.481, t_4_ = 1.431; P_1_ = 0.031, P_2_ = 0.003, P_3_ = 0.167, P_4_ = 0.180)

Y_2_ = 0.416 + 1.395X_2_ + 0.038X_4_ (F = 25.1; R = 0.899, Ry_1_ = 0.511, Ry_2_ = 0.812; t_1_ = 2.058, t_2_ = 4.831; P_1_ = 0.060, P_2_ = 0.003).

The F test results show that the regression equation of Y_1_ reached a very significant level. The significance test of the partial regression coefficient indicated that P and SOM were the most important factors that influenced Cd concentration in soil. Surface Cd concentration increases with an increase in the P and SOM concentrations in soil. The regression equation of Y_2_ did not reach a significant level. Although the concentration of As showed a non-significant linear relationship with SOM and P, As was apparently mainly influenced by SOM and P. Therefore, SOM and P were the key factors for Cd and As enrichment in forest soil in karsts.

### 3.5. Analysis of Cd and As Enrichment Mechanisms

#### 3.5.1. Factors of Geological Background

The concentrations of Cd and As in the study area exceeded the standards for soil environmental quality in China and the corresponding PTE background values in Guizhou Province. The research area is a forest without mining activities and is less affected by human activities. Therefore, the enrichments of both Cd and As were not a result of human factors. We speculate that enrichment could be mainly controlled by the natural environment.

The concentrations of Cd and As in the bedrock were 1.65 and 3.9 times the crustal abundance in the study area. Both values were greater than one, thereby indicating that Cd and As had high background values in carbonate rocks. This finding further indicates that the enrichments of Cd and As in the karst area may be restricted by carbonate lithology [16]. Rambeau et al. [35] argued that the concentration of Cd in Jurassic limestone in the Lower Burgundy region of France reached as high as 2.6 mg/kg, which indicates that Cd enrichment in the karst region is closely related to the high background values of carbonate rocks. He et al. [36] pointed out that the high concentration of Cd in the area was mainly distributed in exposed carbonate rocks. Ni et al. [37] noted the enrichment trend of As in carbonate rocks of the Lower Paleozoic in Beijing by the symbiotic soil covering the rocks. In addition, the results of the 1:200,000 regional geochemical sweeping in Yunnan Province showed that the regions with geochemically high contents of As and Cd had good spatial coupling with widely distributed carbonates [14].

The concentrations of Cd and As in Horizon C of the five rock–soil profiles in the study area were relatively enriched, thereby indicating second-stage enrichment. Previous studies have shown that overlying soils and underlying carbonate rocks in Switzerland clearly have a shared provenance with the underlying carbonate rocks. The high Cd concentration in the soil was closely related to the high concentrations of Cd in the bedrock [38]. In addition, the ratio of Cd to limestone bedrock concentration in the Lower Burgundy region of France was between 4.6 and 5.7, which indicates that soil developed by carbonate weathering exhibits obvious Cd enrichment characteristics [35]. The weathering of carbonate rocks is an important source of Cd and As enrichment for soil in the karst area of Yunnan. The accumulation of As and Cd from rock to soil was approximately 4 to 5 times, and the enrichment mechanism was mainly due to the adsorption of clay minerals and organic matter [39]. A study also showed that soil formed by the weathering of carbonate rocks leads to the relative enrichment characteristics of Fe, Al, Mn, and As compared with the bedrock. Conversely, the concentrations of Ca and Mg are relatively deficient in the bedrock [40].

On the one hand, the high concentrations of Cd and As in soil are related to the high background values of carbonate rocks. On the other hand, this phenomenon may be due to clay mineral enrichment, such as the oxides and hydroxides of Fe and Al, which may cause the enrichment of Cd and As concentrations in the soil parent material compared to the bedrock.

#### 3.5.2. Effects of Plant Growth Processes

The study area is a forest with vigorous vegetation. The Cd and As in the soil absorbed by plants were returned to the surface soil in the form of litter, which provides a source for the accumulation of Cd and As on the soil’s surface [41].

Excess phosphates in the study area may induce the precipitation of Cd_3_(PO4)_2_ and Cd^2+^ [42,43]. The competitive adsorption between P and As in soil can reduce the adsorption of As in soil with an increase in P concentrations [44]. Therefore, Cd in the topsoil is more concentrated than As. The forest area is flourishing, and SOM is largely accumulated under biological actions [45,46]. A large number of studies have shown that SOM has specific complexation and accumulation capacities for heavy metals in soil because of its abundant functional groups [47,48,49]. SOM is an important adsorbent of heavy metals in soil because it can retain heavy metals in soil by forming metal–SOM complexes to reduce the mobility of heavy metals [50]. Lin et al. [51] showed that removing SOMs resulted in an average decrease of 20% in the adsorption of Cd^2+^. Luo et al. [52] pointed out that concentrations of Cd in soil generally decreased with a deepening of stony desertification in a karst, which indicates that the concentration of heavy metals decreased with a decrease in SOM.

Therefore, topsoil Cd and As were mainly accumulated due to the large amount of SOM complexed with heavy metals formed by plant growth and the precipitation of Cd by P and feedback litter.

## 4. Conclusions

We used the enrichment factor, geo-accumulation index, redundancy analysis, and other methods to analyze the enrichment mechanism of Cd and As in the process of rock weathering and soil formation in the karst area. We drew the following conclusions:

(1) The mean concentrations of Cd and As in the lime soil profile were characterized as follows: horizon A > horizon B > horizon C > horizon D. The entire enrichment process was mainly divided into three stages. In the first stage, carbonate was enriched in the Cd and As concentrations relative to the crust. The second stage featured the enrichment of Cd and As in the process of rock weathering and soil formation. The third stage entailed the topsoil enrichment of Cd and As.

(2) The enrichment process and its influencing factors differed across stages. The first stage was mainly influenced by lithology. In the second stage, the adsorption of clay minerals, such as the oxides and hydroxides of Fe and Al, led to the enrichment of Cd and As concentrations in the soil parent material in comparison to the bedrock. Cadimum and As surface aggregation, especially the enrichment of Cd, was more obvious in the third stage. Complexation and precipitation could be the main enrichment mechanisms for Cd and As in topsoil.

(3) This study revealed the enrichment process of soil Cd and As in karst areas. The results could provide a valuable reference for the source apportionment of soil Cd and As in the karstic geochemical anomalous area of Southwest China.

## Figures and Tables

**Table 1:** Geo-accumulation index.

Class	I_geo_ Value	Enrichment level	Class	I_geo_ Value	Enrichment Level
0	I_geo_ ≤ 0	Non-enrichment	4	3 < I_geo_ ≤ 4	Heavy enrichment
1	0 < I_geo_ ≤ 1	Non-enrichment to Moderate enrichment	5	4 < I_geo_ ≤ 5	Heavy to Extreme enrichment
2	1 < I_geo_ ≤ 2	Moderate enrichment	6	I_geo_ > 5	Extreme enrichment
3	2 < I_geo_ ≤ 3	Moderate to heavy enrichment			

**Table 2:** Cadmium (Cd) and Arsenic (As) contents and physicochemical properties of soil.

Topsoil	As	Cd	N	P	HA	SOM	Clay	Slit	Sand	pH
1	21.4	4.09	5.08	0.28	23.17	82.32	13	35	52	7.54
2	26.3	4.27	4.52	0.73	21.31	82.82	19	36	45	7.89
3	25.0	3.94	4.19	0.46	24.52	80.06	27	37	36	7.24
4	30.9	2.71	3.50	0.69	16.86	54.64	19	41	40	7.94
5	36.2	4.03	3.69	0.57	16.55	55.59	24	35	41	7.74
6	29.9	3.06	3.47	0.59	13.55	53.00	26	35	39	7.72
7	26.1	5.92	5.74	1.00	28.65	92.44	14	42	44	7.18
8	24.8	5.23	6.00	0.96	28.17	90.72	11	40	49	7.62
9	22.5	4.29	5.34	0.64	26.69	87.57	10	44	46	7.06
10	20.5	4.13	3.85	0.64	17.38	63.64	29	33	38	7.56
11	19.8	3.98	3.94	0.66	18.41	64.49	17	31	52	7.08
12	22.3	3.25	3.45	0.43	20.27	63.75	15	50	35	7.01
13	17.5	2.84	3.24	0.56	13.86	51.62	20	39	41	7.90
14	18.2	2.67	1.21	0.29	9.62	35.85	25	40	35	7.59
15	14.9	2.97	3.15	0.60	13.24	51.78	18	39	43	8.03
Max	36.2	5.92	6.00	1.00	28.65	92.44	29	50	52	8.03
Min	14.9	2.67	1.21	0.28	9.62	35.85	10	31	35	7.01
Mean	23.8	3.83	4.02	0.61	19.48	67.35	19	38	42	7.54
SD	5.6	0.93	1.20	0.20	5.85	17.39	6	5	6	0.35

Note: The units for As, Cd, N, P, and HA are mg/kg; the unit for SOM is g/kg; the units for Clay, Slit, and Sand are %. N (Nitrogen); P (Phosphorus); HA (Humic acid); SOM (Soil organic matter). Max (Maximum); Min (Minimum); SD (Standard deviation).
